# Binaural Modelling and Spatial Auditory Cue Analysis of 3D-Printed Ears

**DOI:** 10.3390/s21010227

**Published:** 2021-01-01

**Authors:** Te Meng Ting, Nur Syazreen Ahmad, Patrick Goh, Junita Mohamad-Saleh

**Affiliations:** 1 School of Electrical & Electronic Engineering, Universiti Sains Malaysia, Nibong Tebal 14300, Penang, Malaysia; tingtemeng@student.usm.my (T.M.T.); eepatrick@usm.my (P.G.); jms@usm.my (J.M.-S.); 2 Flextronics Systems Sdn. Bhd., Batu Kawan Industrial Park PMT 719 Lingkaran Cassia Selatan, Simpang Ampat 14110, Penang, Malaysia

**Keywords:** 3D-printed ears, binaural modelling, auditory cues, front-rear confusions, 3D localization

## Abstract

In this work, a binaural model resembling the human auditory system was built using a pair of three-dimensional (3D)-printed ears to localize a sound source in both vertical and horizontal directions. An analysis on the proposed model was firstly conducted to study the correlations between the spatial auditory cues and the 3D polar coordinate of the source. Apart from the estimation techniques via interaural and spectral cues, the property from the combined direct and reverberant energy decay curve is also introduced as part of the localization strategy. The preliminary analysis reveals that the latter provides a much more accurate distance estimation when compared to approximations via sound pressure level approach, but is alone not sufficient to disambiguate the front-rear confusions. For vertical localization, it is also shown that the elevation angle can be robustly encoded through the spectral notches. By analysing the strengths and shortcomings of each estimation method, a new algorithm is formulated to localize the sound source which is also further improved by cross-correlating the interaural and spectral cues. The proposed technique has been validated via a series of experiments where the sound source was randomly placed at 30 different locations in an outdoor environment up to a distance of 19 m. Based on the experimental and numerical evaluations, the localization performance has been significantly improved with an average error of 0.5 m from the distance estimation and a considerable reduction of total ambiguous points to 3.3%.

## 1. Introduction

### 1.1. Background

In the field of acoustics and robotics, it would require a minimum of three microphones to triangulate a sound source in a two-dimensional (2D) space [1,2]. With only two omnidirectional microphones, the microphone fields would intersect at two points, implying that there could be two possible locations where the sound could originate from. Having the third microphone will reveal the unique position of the sound source by eliminating the other possibility. Despite only having two ears. i.e., binaural hearing, humans and animals are able to localize a sound source not only in a 2D space, but also in a three-dimensional (3D) space by analyzing different auditory cues. The brain deciphers the audio cues to predict the direction and distance of the sound source [3]. The job of the ears is to capture and send natural acoustic signals to the brain for processing. The shape of the ear and head additionally plays a role in localizing the sound source by reflecting and diffracting the sound to help the brain to identify the direction [4]. Gaming and recording industries have begun using ear shaped recording devices to make binaural recordings giving a more natural hearing experience for the listeners [5]. In the gaming industries, having binaural audio enables the player to identify where a sound source is coming from in the game in order to give the listener a virtual sense of space [6].

Acoustic triangulation is based on the physical phenomena that sound waves are longitudinal when in far field and if the source is not exactly at the center of all microphones, there will be a time delay between the first microphone and subsequent microphones [7]. Specifically in binaural hearing, this is known as the Interaural Time Difference (ITD) [8]. ITD is the difference in the arrival time of a sound between two ears. It is crucial in the localization of sounds, as it provides a cue to the direction or angle of the sound source from the head. The brain would register the time lag and inform the listener of the direction of sound [9]. ITD analysis is one of the techniques used to predict the angle of arrival of the sound source with respect to the receiver on the azimuth plane.

The Interaural Level Difference (ILD) is another spatial auditory cue that helps a human to localize a sound source. ILD is defined as the difference in amplitude between the two ears [10]. When a sound source is closer to one ear, the sound level will be louder in one ear than the other, as sound is attenuated by distance and also the head. The direction of the sound source can be localized by comparing the level difference between the two ears. ITD/ILD is primarily used for low frequency localization under 800 Hz. The head shadow effects increase with frequency, and therefore loudness differences are the primary horizontal localization cues for frequencies above 1500 Hz [11]. In many applications, ILD and ITD are used in tandem for a more accurate position estimation on the horizontal plane.

Vertical localization is essential if one is to estimate the position of a sound source in a 3D space, and for binaural auditory systems, this can only be realized with the existence of the ears. The shape of the ears is in such a way that the amplitude and frequency response changes, depending on where the sound source is located on the azimuth plane. The pinna which is the outer part of the ear acts as a filter to attenuate certain frequency ranges and has a major role in helping the human auditory system to localize the angle and distance of the sound source [12]. Since the shape of the pinna is very complex and asymmetrical, different pinna resonances become active both vertically and horizontally, depending on the location of the source. These resonances add direction-specific patterns into the frequency response of the ears, which is then recognized by the auditory system for direction localization [13].

### 1.2. Related Work, Motivation and Contributions

In a binaural system, there exists a set of points that are equidistant from left and right ears, which results in ILD values that are almost identical, and creating a zone called the cone of confusion. This sound ambiguity that is typically referred to as “front-rear” confusion commonly occurs when localizing a sound source with binaural audition, where it is difficult to determine whether the source is behind or in the front of the receiver [14]. One way to resolve this is by introducing dummy heads, such as the Head and Torso Simulator (HATS) with a microphone placed inside each ear canal to allow for the creation of various acoustic scenes [6]. Via this strategy, several researchers proposed Head-Related Transfer Function (HRTF) estimations, where the transfer functions that are based on left and right ears were preliminarily analyzed and constructed before the binaural model is reproduced [15]. However, some techniques via HRTF have been experimentally outperformed via another approach in [16], which used artificial pinnae made with silicone in order to remove the ambiguity by comparing the mean intensity of the sound source signal in a specific frequency range using a threshold value.

For binaural localization in targeted rooms, statistical relationships between sound signals and room transfer functions can be analyzed prior to real-time location estimations, such as the work presented in [17]. The accuracy can be further enhanced by jointly estimating the azimuth and the distance of binaural signals using artificial neural network [18,19]. Another approach utilizing the room’s reverberation properties has been proposed in [20], where the reverberation weighting is used to separately attenuate the early and late reverberations while preserving the interaural cues. This allows the direct-to-reverberant (DRR) energy ratio to be calculated, which contains the information for performing absolute distance measurement [21].

The interest in most of the aforementioned work is nonetheless gravitated to sound source localization and disambiguation on the horizontal or azimuth plane, with greater focus on indoor environments. In order to estimate the vertical direction of the sound source, spectral cue analysis is required, as the direction heavily depends on the spectral composition of the sound. The shoulders, head, and especially pinnae act as filters interfering with incident sound waves by reflection, diffraction, and absorption [22,23]. Careful selections on the head dampening factor, materials for the ears/pinnae development, and location of the microphones are equally important for a realistic distortion of the sounds [24]. In [25], artificial human ears that were made from silicone were used to capture binaural and spectral cues for localization in both azimuth and elevation planes. To enhance the localization and disambiguation performance while retaining the binaural hearing technique and structure, a number of recent works have proposed using active ears, which is inspired by animals, such as bats, which are able to change the shape of their pinnae [26,27,28]. In this regard, the ears act as actuators that can induce dynamic binaural cues for a better prediction.

While reproducing the auditory model of binaural hearing may be a challenging problem, the past decade has seen a renewed interest in binaural approaches to sound localization, which has been applied in a wide area of research and development, including rescue and surveillance robots, animal acoustics, as well as human robot interactions [29,30,31,32,33,34,35]. Unique or predetermined sound sources for instance can be embedded with search and rescue robots for ad-hoc localization in hazardous or cluttered environments, as well as for emergency signals in remote or unknown areas [36,37]. This approach is particularly useful when searching that is based on visual is occluded by obstacles, but allows for sound to pass through [38].

Inspired by the intricacies of human ears and how they can benefit plethora of applications if successfully reproduced, this research aims to build a binaural model that is similar to the human auditory system for both vertical and horizontal localizations using a pair of ears that were 3D-printed out of Polylactic Acid (PLA). Unlike silicones, which were mostly used by past researchers for binaural modelling [39], PLA is generally more rigid and a more common material in domestic 3D printers. Using 3D printed ears with PLA will also allow for cheaper and quicker replication of this work in future studies. The ears that were anatomically modeled after an average human ear were additionally mounted on a Styrofoam head of a mannequin to get the shape and size of an average human. The purpose is to build a binaural recording system similar to the HATS to be able to capture all different auditory cues, and for the system to have a head shadow to change the spectral components. The HATS replica may not be as good as the actual simulator, but it provides a cheap and quick alternative for simple measurements.

In this work, an analysis on the proposed model was firstly conducted to study the correlations between the spatial auditory cues and the 3D polar coordinate (i.e., distance, azimuth and elevation angles) of the targeted sound source. Apart from the techniques via interaural and spectral cues, the time for the sound pressure level (SPL) resulting from the combined direct and reverberant intensities to decay by 60 dB (hereafter denoted as $DRT_{60}$) is also introduced as part of the localization strategy. The preliminary analysis reveals that the latter provides a much more accurate distance estimation as compared to the SPL approach, but is alone not sufficient to disambiguate the front-rear confusions. For vertical localization, it is also shown that the elevation angle can be robustly encoded through the spectral notches. By analysing the strengths and shortcomings of each estimation method, a new algorithm is formulated in order to localize the sound source, which is also further improved with induced secondary cues via cross-correlation between the interaural and spectral cues. The contributions of this paper can thus be summarized as follows:

(a) auditory cue analysis of ears that were 3D-printed out of cheap off-the-shelf materials, which remains underexplored; and,
(b) a computationally less taxing binaural localization strategy with $DRT_{60}$ and improved disambiguation mechanism (via the induced secondary cues)

This work is motivated by recent studies on binaural localizations for indoor environments that utilized ITD in a 3D space [40], both ILD and ITD [41] in a 2D space, and DRR for distance estimation of up to 3 m [42]. The aforementioned literatures however did not use pinnae for front-rear disambiguation, hence requiring either a servo system to integrate rotational and translational movements of the receiver or other algorithms to solve for unobservable states in the front-rear confusion areas until the source can be correctly localized. Nevertheless, instead of targeting indoor environments with additional control systems to reduce the estimation errors, the focus of this work is on outdoor environment with a relatively larger 3D space. As both the DRR and reverberation time (RT) change with the distance between the source and the receiver [43], particularly in outdoor spaces [44,45], this property has been exploited and correlated with the distance to further improve the estimation accuracy. The proposed technique in this study has been validated via a series of experiments where the sound source was randomly placed at 30 different locations with the distance between the source and receiver of up to 19 m.

## 2. Sound Ambiguity

**Figure 1:**
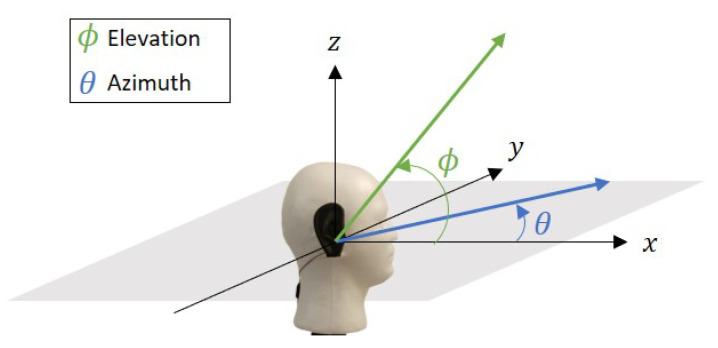
Illustration on the azimuth angle, θ, and elevation angle, ϕ, with respect to the model in three-dimensional (3D) space.

Human binaural hearing is able to approximate the location of a sound source in a spherical or 3D coordinate (i.e., azimuth and elevation planes). This is achieved by the shape and position of the ears on the head, and the auditory cues interpreted by the brain. As illustrated in Figure 1, the azimuth angle is represented by θ, while the elevation angle is represented by ϕ. Moving the sound source from left to right would change θ and moving it up and down will change ϕ, and each varies from 0° to 360°.

**Figure 2:**
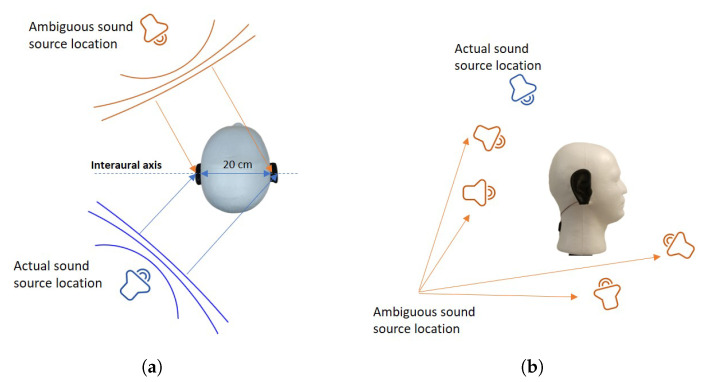
(**a**) Ambiguity on the azimuth plane; (**b**) ambiguity on the elevation plane.

With only two microphones in a binaural system, there will be localization ambiguity on both azimuth and elevation planes. With regard to the azimuth plane, for every measurement, there will be an ambiguous data point located at the mirrored position along the interaural axis where the two microphones are placed as illustrated in Figure 2a. Localizing the sound source on the elevation plane is relatively more difficult as there will be an infinite amount of ambiguous positions as depicted in Figure 2b. This paper looks into finding the actual sound location by using auditory cues. When the distance between a sound source and the microphones is significantly greater than the distance between the microphones, we can consider the sound as a plane wave and the sound incidence reaching each microphone as parallel incidence.

For the elevation angle ϕ, when the sound source is located at θ=0°, ITD and ILD for each ear would theoretically be the same. For an omni directional microphone, this would be impossible to solve as there are infinite number of possibilities where the actual sound source could be located, as there would be no difference in values when taking measurements. Nevertheless, with the addition of the head and ear, sound is reflected and attenuated differently, as it is moved around the head. Attenuation happens in both the time and frequency domain for different frequency ranges. This work aims to localize a sound source in the azimuth and elevation planes while only retaining the actual location by removing the ambiguous points. The proposed method in this work is based on the analysis of different auditory cues and characterization of their properties in order to estimate the location of the sound source relative to the receiver.

## 3. Materials, Methods and Analysis

**Figure 3:**
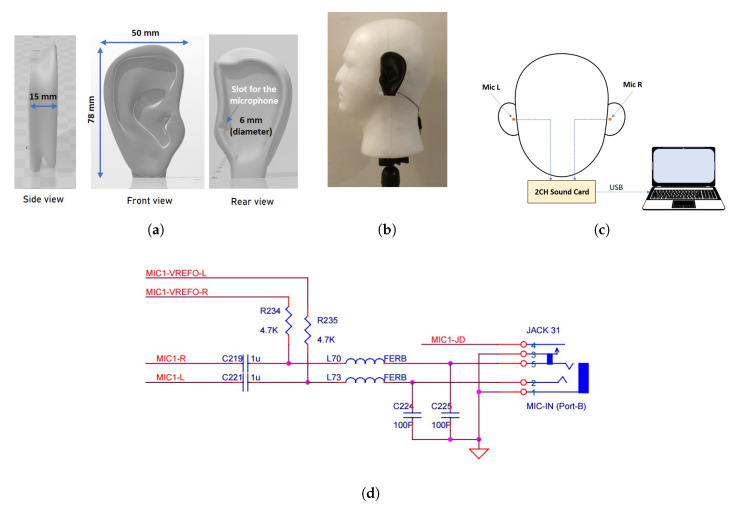
(**a**) Illustrations on the ear model from the STL file; (**b**) Left view of the HATS with the 3D printed ear; (**c**) Sketch of the setup with microphones on the left and right ears (i.e., Mic L and Mic R). (**d**) Detailed connections between the microphones and the computer.

The experiment setup in this work follows the HATS, where the geometry is the same as an average adult. For this work, the ear model which was 3D-printed out of PLA was scaled to the dimensions, as shown in Figure 3a to fit the application (for the pinnae shape, we have referred to https://pubmed.ncbi.nlm.nih.gov/18835852/ which provides the database containing 3D files for human parts for biomedical research). A microphone slot for each side of the head model was also designed as depicted in Figure 3a,b, and the two microphones were connected to a two-channel sound card (Focusrite Scarlett 2i2 model) for simultaneous recording (Figure 3c). The hardware component consists of two parts, namely the microphone bias and the sound card, as shown in Figure 3d. The gain was adjusted for each ear to balance the gain for the left and right ear. The ears were also polished with a solvent to smooth out the plastic parts, and treated with the fumes from the solvent to smooth out the internal parts. A mechanical mesh was then placed on top of each microphone when assembling the 3D printed ear to act as a filter. For printing, a DreamMaker Overlord 3D printer was used. Details on the printing parameters are listed in Table 1 (the printer is available at https://www.dfrobot.com/product-1299.html, while the STL file is available from the Supplementary Materials). The total cost for this setup is approximately USD175 (i.e., USD11.4 for the 3D-printed ears, USD 2.89 for the Styrofoam head, USD156.5 for the sound card, and USD 4.2 for the bias circuit).

**Figure 4:**
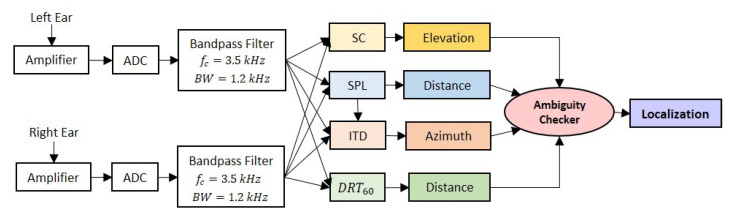
Binaural processing chain within the device under test (DUT) to localize the sound source where ’$DRT_{60}$’, ’SC’, ’SPL’, and ’ITD’ refer to the auditory cues.

Figure 4 shows the binaural processing chain within the device under test (DUT) in order to localize the sound source. The first stage is the data acquisition from the left and right inputs of the microphones. To ensure no sound leakage, the microphone is sealed to the printed ear with silicone. The next stage is the amplifier stage, which biases the signal to 1.5 V. The microphones used are rated to 3.0 V, so using a standard lithium battery was enough to prevent the microphones from saturating at the reference point. Following the amplifier (after the analog-to-digital (ADC) converter) is the filtering stage, which consists of a bandpass filter with a cut-off frequency, $f_{c}=3.5$ kHz and a bandwidth, BW = 1.2 kHz to attenuate the effects from environmental noise. The sound source considered has a frequency range from 2.8 kHz to 4.0 kHz, hence other frequencies beyond this range can be filtered out.

The following block is the analysis of the auditory cues split into four categories, spectral cues (SC), and $DRT_{60}$ (explained further in Section 3.2), SPL and ITD. Fast Fourier Transform (FFT) is performed on the filtered signal in order to find the spectral components in the frequency domain of the audio source used in SC. $DRT_{60}$, SPL, and ITD, on the other hand, are measured in time domain. The ITD and SC are essential for azimuth angle and elevation angle estimations respectively. In order to estimate the Euclidean distance between the center of the DUT and the sound source, both $DRT_{60}$ and SPL will be used. Ambiguous data points are then filtered before the sound source is localized.

**Figure 5:**
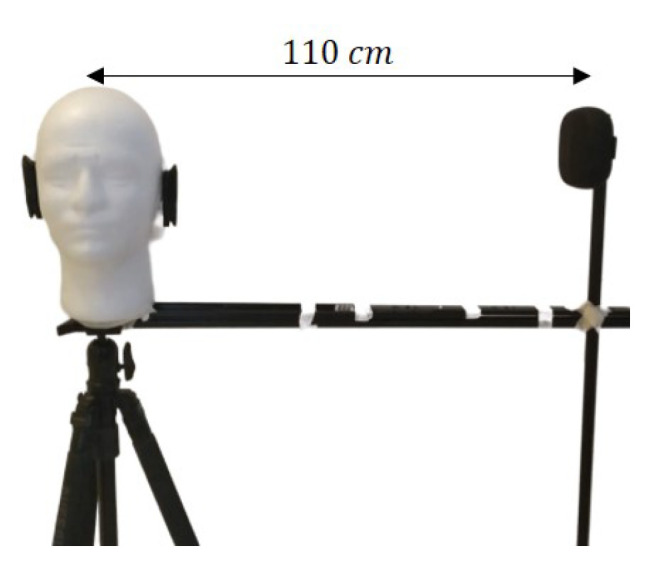
A Bluetooth speaker placed at 110 cm was rotated around the model during the measurements.

**Figure 6:**
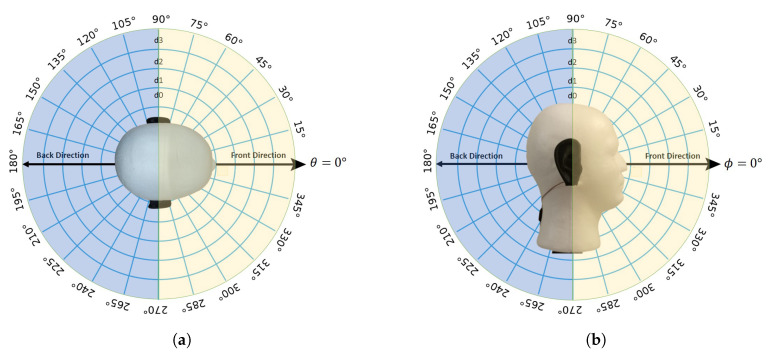
Illustration on (**a**) θ on the azimuth plane; and (**b**) ϕ on the elevation plane.

Before the actual experiment was conducted, a preliminary analysis was performed in order to ensure its feasibility. To observe the SPL and frequency responses with respect to azimuth and elevation angles, a sound source was placed at d=110 cm from the receiver and positioned on a rotating jig as depicted in Figure 5. A Bluetooth speaker was used in order to play a recording of the sound source intended for the actual experiment. Figure 6 shows how θ and ϕ are measured on their respective planes.

**Figure 7:**
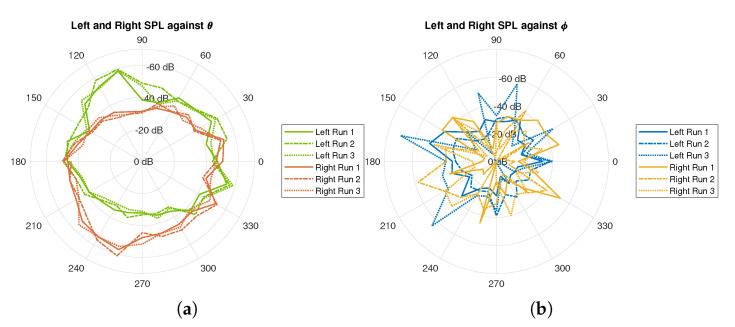
Sound pressure level (SPL) measured from the three trials for right and left ears on (**a**) azimuth plane; and, (**b**) elevation plane.

**Figure 8:**
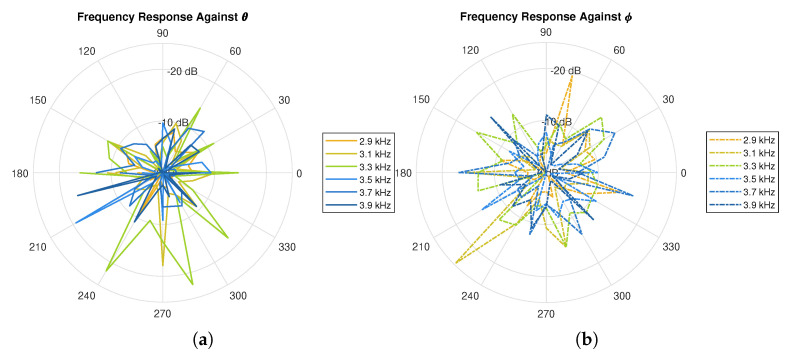
Frequency responses on (**a**) azimuth plane, and; (**b**) elevation plane.

During the pilot testing (a controlled environment with a noise floor below −60 dB was selected for this testing), the sound source was rotated along the azimuth and elevation planes at a step of 15°. The jig was used to ensure consistent angle increments and keep the sound source at a fixed distance. In this test, both left and right audio were captured simultaneously, and each test was repeated three times to analyze the consistency of the measurement setup. Figure 7 shows the polar plots for the SPL that was measured at the left and right ears for the three trials on both the azimuth and elevation planes. For every instance of azimuth and elevation angle, FFT was applied to the signal and the peak at each desired frequency point was measured. Figure 8 illustrates the frequency responses of the spectral components of the sound source.

**Figure 9:**
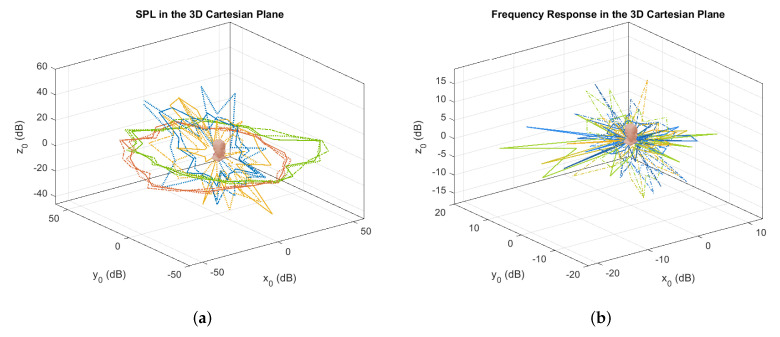
Illustrations of SPL (**a**) and frequency response (**b**) in the 3D Cartesian plane relative to the DUT (represented by the head icon).

Figure 9 illustrates the variations of the SPL and the frequency response in a 3D Cartesian plane, where $x_{0},y_{0}$ and $z_{0}$ correspond to dcosθ, dsinθ and dsinϕ, respectively. Based on the SPL response, it is observed that the variations of the amplitude are relatively much smaller on the azimuth plane (i.e., *y* vs *x*) as compared to that on the elevation plane (i.e., *z* vs *y*). With regard to the frequency response, it can be seen that the amplitude on the azimuth and elevation planes change significantly enough that it is distinguishable from other coordinates. This is due to the shape of the ears as well as reflections around the head which induced notches into the spectrum. This signifies the suitability of the cues to be used as part of the techniques for horizontal localization, and the notches in the frequency response for vertical localization. The following sections will describe in greater detail how these properties, along with ITD, $DRT_{60}$, and SC will be exploited in order to localize the sound source.

### 3.1. Interaural Time Difference (ITD)

**Figure 10:**
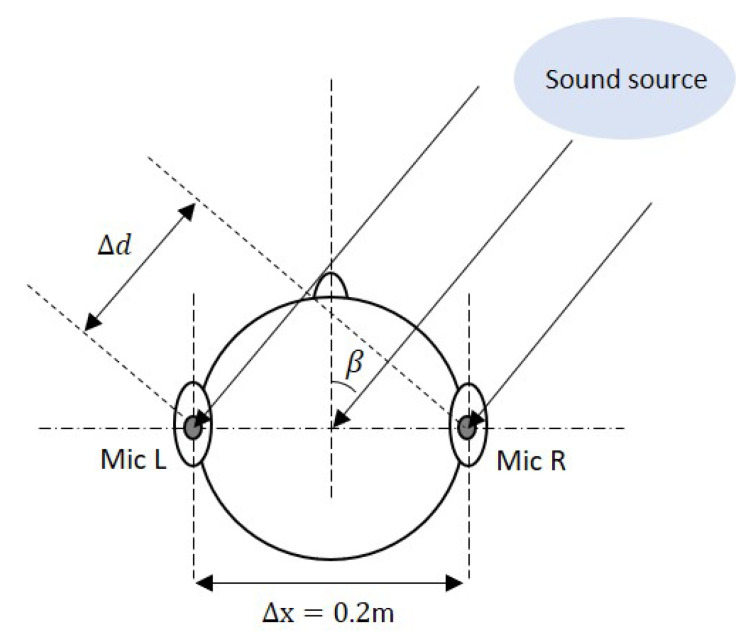
Illustration of angle of arrival (AoA) calculation (not to scale).

In order to estimate the direction of the sound source, the angle of the incident wave with respect to the DUT, which is also known as angle of arrival (AoA) needs to be found. This is done by comparing the delay between the sound signal of the two microphones, which is termed ITD in the context of binaural hearing. To this purpose, let the ITD be written as $\tau_{d}=\left|t_{R}-t_{L}\right|$, where $t_{R}$ and $t_{L}$ refer to the time of arrival of the sound between both microphones, which is 0.20 m, and $\nu_{s}$ is the speed of sound, i.e., 343 ms${}^{-1}$. From the illustration shown in Figure 10, it can be intuitively seen that the wave front will arrive at Mic L later than it does at Mic R. The AoA, β, as seen by Mic L relative to Mic R can be calculated using Equation (1), below.

(1) $$\Delta d=\nu_{s}\tau_{d};\;\Delta d=\Delta x\sin\beta ;\;\beta =\arcsin\left(\frac{\Delta d}{\Delta x}\right).$$

To quantify the phase shift, cross-correlation was applied in order to measure the ITD between the two signals. The cross-correlation in Equation (2) is used to calculate the ITD between the two signals, where N=44,100 refers the total number of observations, $m_{a}\left(i\right)$ is the signal that is received by Mic R and $m_{b}\left(i\right)$ is the signal received by Mic L. The notations ${\bar{m}}_{a}$ and ${\bar{m}}_{b}$ denote the mean of $m_{a}\left(i\right)$ and $m_{b}\left(i\right)$, respectively. The cross correlation coefficient $R_{ab}$ can then be calculated, as follows

(2) $$R_{ab}\left(\tau_{d}\right)=\frac{\sum_{i=1}^{N}\left[\left(m_{a}\left(i\right)-{\bar{m}}_{a}\right)\cdot \left(m_{b}\left(i-\tau_{d}\right)-{\bar{m}}_{b}\right)\right]}{\sqrt{\sum_{i=1}^{N}(m_{a}\left(i\right)-{\bar{m}}_{a}}{)}^{2}\sqrt{\sum_{i=1}^{N}{\left(m_{b}\left(i-\tau_{d}\right)-{\bar{m}}_{b}\right)}^{2}}}$$

which would return a value ranging from −1 to 1. Audio was taken at a sampling rate, $f_{s}$, of 44,100 samples per second. The returned value of the cross correlation coefficient would denote how many samples apart the two wave forms are.

It is worth noting that the ILD can also be used as a means of measuring the AoA by comparing the ratio of attenuation between each ear. The amount of attenuation and the ratio between left and right would be characterized by placing the sound source at θ=90° and θ=270°. The ILD is able to capture the AoA by comparing the attenuation, but it is not as accurate as using the ITD. As an example, when the audio source is closer to the left ear at θ=45°, the amplitude is higher than the right and vice versa. When the sound source is at θ=0°, the amplitude is roughly the same level. The method of using ILD to estimate the angle is inaccurate and unreliable when compared to cross correlation of ITD. There are many factors affecting the attenuation of sound, such as environment, distance from sound source, and reflection, which can cause the estimation of angle based on this parameter to be temperamental. Since cross correlation looks at the similarity of the audio signal between left and right, it is more robust and not as susceptible to interference. In this work, the cross correlation of ITD is more consistent at determining the AoA when compared to the attenuation ratio method of estimating AoA based on ILD. From the testing, the ILD estimation method using the attenuation ratio has an error of ±20°, while ITD has an error of ±10°. Although the ILD is not directly used in the estimation of angle in this study, the SPL at each ear are instrumental for distance estimation and front-rear disambiguation. The subsequent sections will present the analyses on $DRT_{60}$ and ILD along with the proposed methods in order to estimate the distance and direction of the sound source.

### 3.2. Direct and Reverberant Energy Fields

While the RT is predicted to be constant using the Sabine’s equation in many enclosed acoustical environments, it has been shown in [43] that it can vary with the distance between the sound source and the receiver under certain circumstances, thus contributing to the variation of the DRR with distance. The dependency of the RT with distance is also more prominent in outdoor spaces as reported in [44,45]. As a consequence, the SPL that is measured at the receiver is usually a combination of energies from both the direct and reverberant fields, which is consistent with the theoretical conclusion in [21]. Hence, depending on applications, considering the combined pressure level would be relatively more practical due to the observed dynamics of both DRR and RT in past studies.

**Figure 11:**
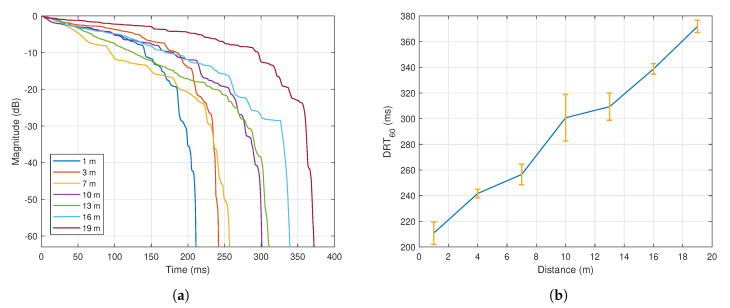
(**a**) Time response of the sound amplitude at θ=0°,ϕ=0° after the sound source was abruptly switched off at varying distances; (**b**) $DRT_{60}$ against distance—blue line denotes the average values for five trials, while vertical yellow line denotes the corresponding error bar.

In this work, a car honk was used as the targeted sound source as it creates distinctive acoustic characteristics that are suitable for outdoor spaces. The impulse to noise ratio (INR) for this sound is above 44.2 dB, which is sufficient according to the ISO 3382-2 for accurate RT measurement in outdoor spaces within 50 m range [45]. Its unique identity was represented by its frequency components, where the range varied from 2.9 kHz to 4.0 kHz with peaks at every 200 Hz interval. In this analysis where the setup was done outdoors, the sound source was initially placed at the front of the DUT on the azimuth plane (i.e., θ=0°,ϕ=0°), and data was captured when it was located at varying distances ranging from 1 m to 19 m. Figure 11a shows the time response of the measured sound amplitude after the source was abruptly switched off at different distances. In order to calculate the $DRT_{60}$, which refers to the time for the combined direct and reverberant energy level to decay by 60 dB, the perceived signal was firstly band-passed to the desired frequency range of 2–4 kHz. Considering $E\left(t\right)=\int_{t}^{\infty }h^{2}\left(\tau \right)d\tau$ as the energy decay curve from time *t* where h(t) is the impulse response from the band-passed signal, a linear regression was performed in order to estimate the slope, S, between the −5 dB and −25 dB level range (similar to RT estimation via T20 method: https://www.acoustics-engineering.com/files/TN007.pdf). The $DRT_{60}$ can then be estimated as −60/S. The corresponding $DRT_{60}$ against distance is depicted in Figure 11b which shows the average $DRT_{60}$ of five trials along with the error bars.

**Figure 12:**
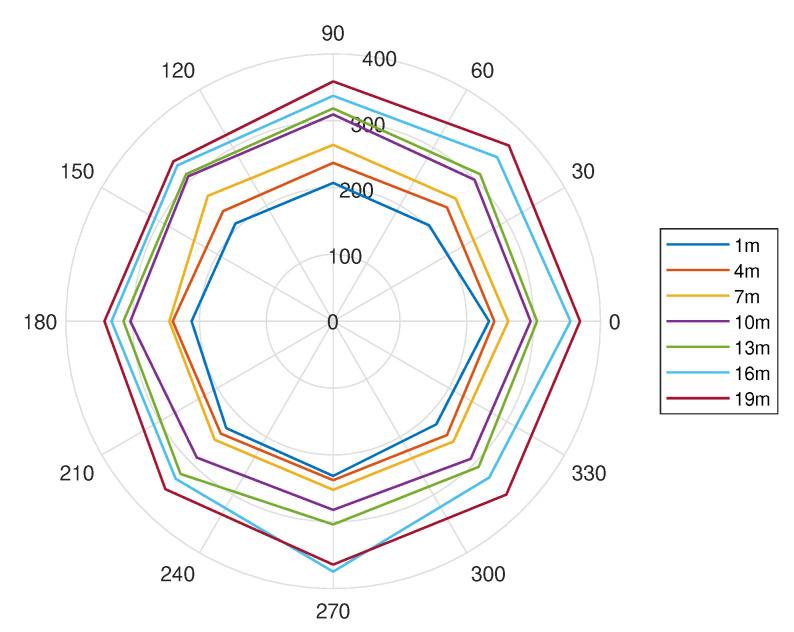
$DRT_{60}$ against θ at varying distances.

In order to analyze the variation of $DRT_{60}$ further, the same test was conducted with θ varied from θ=0° until θ=360° at a step of 45°. Figure 12 shows the $DRT_{60}$ against the azimuth angle. The measured $DRT_{60}$ however did not reveal any distinctive trend and it only has small deviations at different angles.

Comparing Figure 11 and Figure 12, it can be concluded that the $DRT_{60}$ value changes most significantly against distance, and the variation against θ is negligibly small. The next section will explain how the $DRT_{60}$ response will be used along with the SPL in order to estimate the distance and treat the ambiguity issue.

### 3.3. Ambiguity Elimination and Distance Estimation

**Figure 13:**
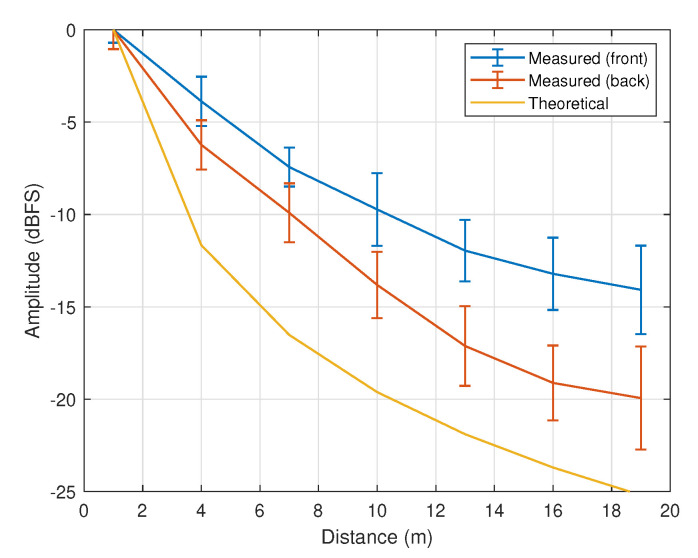
Measured amplitude against distance when θ=0° (front) and when θ=180° (back). The theoretical curve based on the inverse square law is represented by the yellow line.

Apart from the $DRT_{60}$ test, another test to investigate the variation of SPL was also conducted. Figure 13 shows the variations of the average SPL from both ears against distance when the sound source was located at the front (blue line) and back (orange line) positions. The average amplitude and error bars are represented by the curve and vertical lines, respectively. Theoretically, the sound intensity changes with distance following the inverse square law, as represented by the yellow line in the figure.

A large difference can be seen from the theoretical and measured SPL curves due to the existence of ears and head as well as environmental effects. The amplitude attenuation is also relatively higher when the sound source is located at the back of the head as compared to the front. Based on the SPL measurements, the following correlation can be derived:

(3) $$\begin{array}{c} \alpha_{j}\left(d\right)=p_{j}d^{q_{j}}+r_{j};\;p_{j}\in R^{-}\;q{,}_{j},r_{j}\in R^{+}\\ 0<d\leq 30;\;-30<\alpha_{j}<0;\;\mathrm{for}\;j=f,b; \end{array}$$

where $\alpha_{f}$ and $\alpha_{b}$ represent the average SPL for front and back positions respectively. Via curve fitting techniques, one will obtain $\left(p_{f},q_{f},r_{f}\right)=\left(-5.2,0.4689,7.085\right)$ and $\left(p_{b},q_{b},r_{b}\right)=\left(-7.7,0.4599,19.989\right)$. It is worth noting that the sound amplitude alone is insufficient to determine both the distance and direction. To treat this issue, the attenuation of the sound source is used in order to eliminate the ambiguity of sound’s location, since the $DRT_{60}$ value is relatively more consistent for all values of θ and ϕ. Via regression, Equation (4), which provides a less mean squared error than other polynomials can be derived with $\left(p_{R},q_{R},r_{R}\right)=\left(0.01693,8.3494,204.1312\right)$, which represents the correlation between $DRT_{60}$ (denoted by $\tau_{R}$ in milliseconds) and the distance *d*.

(4) $$\begin{array}{cc} \tau_{R}\left(d\right) & =p_{R}d^{2}+q_{R}d+r_{R};\;p_{R},q_{R},r_{R},\in R^{+}\\ \; & 0<d\leq 30;\;100<\tau_{R}<400. \end{array}$$

Hence, the inverse function of Equation (4) can be attained as follows:

(5) $$\begin{array}{cc} d_{R} & =-0.5q_{R}/p_{R}+\left(0.5/p_{R}\right)\sqrt{q_{R}^{2}-4p_{r}\left(r_{R}-\tau_{R}\right)} \end{array}$$

which returns the distance estimated based on the value of $\tau_{R}$ measured from the received signal.

**Figure 14:**
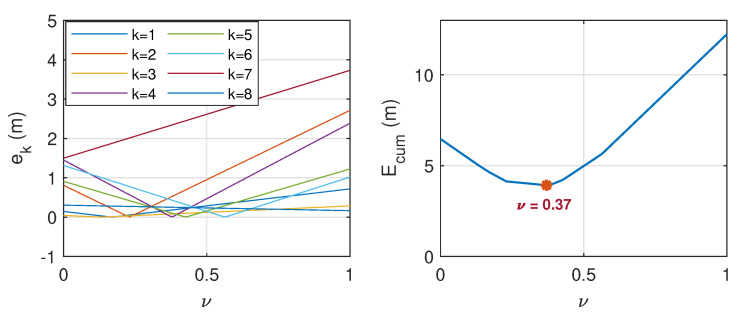
Distance error, $e_{k}$ (**left**) and cumulative distance error, $E_{cum}$, (**right**) when $\phi \in \Omega_{f}$. $E_{cum}$ is minimum when ν=0.37.

**Figure 15:**
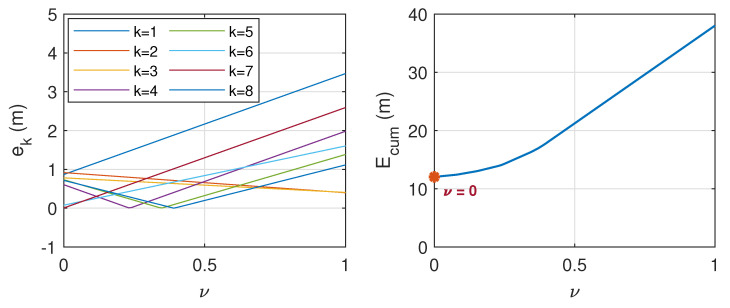
Distance error, $e_{k}$ (**left**) and cumulative distance error, $E_{cum}$, (**right**) when $\phi \in \Omega_{b}$. $E_{cum}$ is minimum when ν=0.

Likewise, the estimated distance based on SPL measurements can be obtained in a similar manner from Equation (3), which leads to   

(6) $$\begin{array}{c} d_{j}={\left(\left(\alpha -r_{j}\right)/p_{j}\right)}^{\left(1/q_{j}\right)};\;j=f,b \end{array}$$

where α is the SPL, $d_{f}$ and $d_{b}$ are the predicted distance values for the front and back locations. In order to eliminate the sound source ambiguity, two parameters need to be observed; the first one is the difference between $d_{j}$ and $d_{R}$, and the second one is the elevation angle ϕ (the method to estimate this is presented in Section 3.4). For the first one, the values of $d_{b}$ and $d_{f}$ are compared against the value of $d_{R}$, and the one with the closer value will return the estimated distance and direction based on SPL, denoted by $d_{\alpha }$, and the other will be the ambiguity to be eliminated. With regard to the second parameter, two sets of angles can be firstly defined, as follows:

(7) $$\Omega_{f}=\left\{\phi \in R\;|\;\phi \in \left[0{}^{\circ},90{}^{\circ}\right]\cup \left(270{}^{\circ},360{}^{\circ}\right]\right\};\;\mathrm{and}\;\Omega_{b}=\left\{\phi \in R\;|\;\phi \in (90{}^{\circ},270{}^{\circ}]\right\}$$

where $\Omega_{f}$ and $\Omega_{b}$ refer to the yellow area and blue area in Figure 6b, respectively. The ambiguity checker can then be written as:

(8) $$\left(d_{\alpha },\eta \right)=\left\{\begin{array}{cc} \left(d_{f},1\right) & \mathrm{if}\;\;\left\{d_{bR}>d_{fR}\right\}\cap \left\{\phi \in \Omega_{f}\right\}\\ \left(d_{b},0\right) & \mathrm{if}\;\;\left\{d_{bR}\leq d_{fR}\right\}\cup \left\{\phi \in \Omega_{b}\right\} \end{array}\right.$$

where $d_{bR}=\left|d_{b}-d_{R}\right|$, $d_{fR}=\left|d_{f}-d_{R}\right|$, and η=1 and η=0 indicate whether the sound source is located at the front position with respect to DUT respectively. As there are now two methods for estimating the value of *d* (i.e., via $DRT_{60}$ and via SPL), the following technique is proposed:

(9) $$\hat{d}=\nu d_{\alpha }+\left(1-\nu \right)d_{R};\;\nu \in \left[0,1\right]$$

with ν representing the weighting parameter that varies between 0 and 1. To find the optimal value of ν, a further analysis was conducted based on 16 datasets, as presented in Table A1 (in Appendix A), where half of them refer to the case when $\phi \in \Omega_{f}$, while the other half refer to the case when $\phi \in \Omega_{b}$. In this analysis, the distance between the DUT and source varied between 6 m and 19 m. The cumulative distance error, which reads:

(10) $$E_{cum}=\sum_{k=1}^{8}e_{k};\;e_{k}=\left|d-\hat{d}\right|$$

with *d* being the actual distance is considered. Figure 14 and Figure 15 show the corresponding plots when $\phi \in \Omega_{f}$ and when $\phi \in \Omega_{b}$, respectively. By observing the value of ν when $E_{cum}$ is minimum, it is found that the distance error can be minimized when ν=0.37 when $d_{\alpha }=d_{f}$, and ν=0 when $d_{\alpha }=d_{b}$. The latter indicates that the estimated distance that is based on the $DRT_{60}$ is generally much closer to the actual value when the sound source is located at the back of the DUT, thus only $d_{R}$ is considered in this scenario.

Combining Equations (8) and (9) and solutions from Figure 14 and Figure 15, the distance estimation along with ambiguity elimination can be further derived, as follows:

(11) $$\begin{array}{c} \hat{d}=\left\{\begin{array}{cc} 0.36d_{f}+0.64d_{R} & \mathrm{if}\;\;\epsilon =1\\ \;\;d_{R} & \mathrm{if}\;\;\epsilon =0 \end{array}\right. \end{array}$$

where

(12) $$\epsilon =\left\{\begin{array}{cc} 1 & \mathrm{if}\;\;\left\{d_{bR}>d_{fR}\right\}\cap \left\{\phi \in \Omega_{f}\right\}\\ 0 & \mathrm{if}\;\;\left\{d_{bR}\leq d_{fR}\right\}\cup \left\{\phi \in \Omega_{b}\right\} \end{array}\right.$$

### 3.4. Spectral Cues (SC)

**Figure 16:**
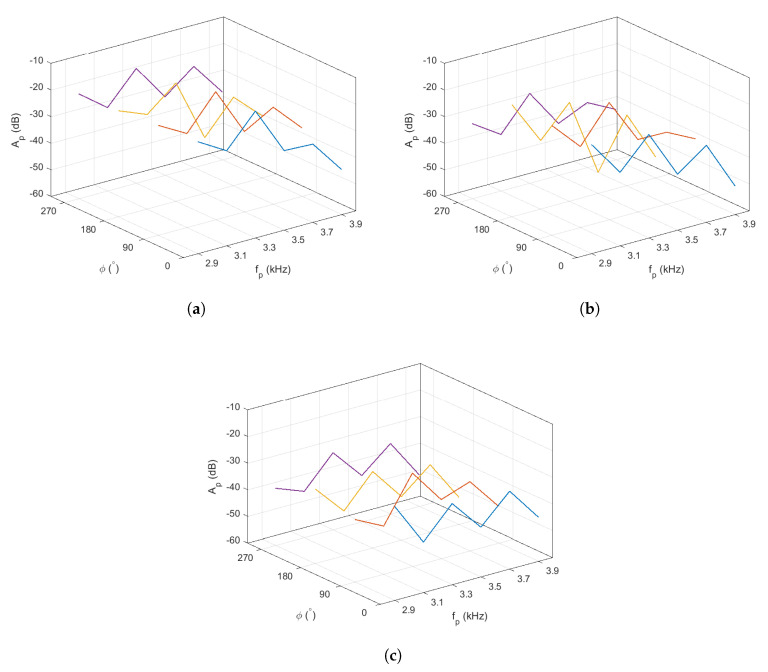
Illustrations on the frequency response at varying ϕ when (**a**) d=6 m; (**b**) d=13 m; (**c**) d=19 m. The z-axis denotes the average amplitude ($A_{p}$) at the peak frequency $f_{p}$. The blue, orange, yellow and purple lines denote the $A_{p}$ at ϕ=0°, ϕ=90°, ϕ=180°, and ϕ=270° respectively.

The clues to sound location that come from sound frequency are called spectral cues. These spectral cues derive from the acoustical filtering of an individual’s auditory periphery. Since the angle and distance on the azimuth plane can be calculated using ITD, SPL and $DRT_{60}$, but not for the elevation plane ϕ, the spectral cues are vital in determining the elevation of the sound source. The ambiguous data points in the cone of confusion can be reduced using mathematical estimation. This work addresses the cone of confusion by characterizing the attenuation of different frequency elements against ϕ. Figure 16 depicts the amplitude ($A_{p}$) at each peak frequency, $f_{p}$, when the sound source was placed at ϕ=0° (blue line), ϕ=90° (orange line), ϕ=180° (yellow line), and ϕ=270° (purple line). The data were also captured at three different distances; d=6 m (a), d=13 m (b), and d=19 m (c).

In order to characterize the amplitude response against ϕ at each peak frequency, a linear regression was performed based on the average values of $A_{p}$ in Figure 16, which led to the following statement:

(13) $$A_{p}=\left\{\begin{array}{cc} \gamma_{1}\left(\phi \right) & \mathrm{if}\;f_{p}=2.9\;kHz\\ \gamma_{2}\left(\phi \right) & \mathrm{if}\;f_{p}=3.1\;kHz\\ \gamma_{3}\left(\phi \right) & \mathrm{if}\;f_{p}=3.3\;kHz\\ \gamma_{4}\left(\phi \right) & \mathrm{if}\;f_{p}=3.5\;kHz\\ \gamma_{5}\left(\phi \right) & \mathrm{if}\;f_{p}=3.7\;kHz\\ \gamma_{6}\left(\phi \right) & \mathrm{if}\;f_{p}=3.9\;kHz\\ \mathrm{undefined} & \mathrm{otherwise} \end{array}\right.$$

where

(14) $$\begin{array}{c} \gamma_{i}\left(\phi \right)=a_{i}\phi^{2}+b_{i}\phi +c_{i}\alpha_{0},\;a_{i},c_{i}\in R^{+},\;b_{i}\in R^{-};\;i=1,2,\ldots ,6, \end{array}$$

with $\alpha_{0}\in R^{-}$ being the amplitude in dBFS of the received signal, and $\alpha_{i},c_{i}\in R^{+}$ and $b_{i}\in R^{-}$ are the coefficients that depend on $\alpha_{0}$.

By measuring $A_{p}$ and $\alpha_{0}$ from the incoming signals’ spectral components, the angle $\phi_{i}$ can then be calculated by solving the inverse function of Equation (14), which reduces to

(15) $$\phi_{i}=-0.5b_{i}/a_{i}-\left(0.5/a_{i}\right)\sqrt{b_{i}^{2}-4a_{j}\left(c_{i}\alpha_{0}-\gamma_{i}\right)};\;i=1,2,\ldots ,6.$$

**Figure 17:**
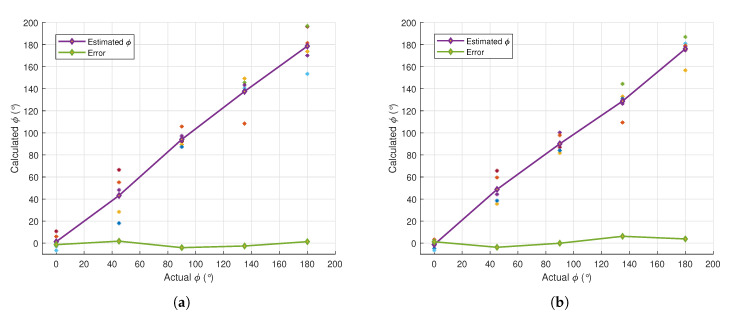
Estimated elevation angle, ϕ, based on SC when the sound source was placed at a distance of 6 m (**a**) and 13 m (**b**) from the DUT. The green line corresponds to the error, while the scatter plots correspond to the values of $\phi_{i}$ at different peak frequencies.

In order to obtain the estimated ϕ when the sound source is placed at a particular location, the calculated angle is averaged over all peak frequencies. Figure 17 shows the results from a simple test when the source was placed at ϕ=(0°,45°,90°,135°,180°). The left plot is the case when the source was 6 m away from the DUT, while the right plot is the case when the source was 13 m away from the DUT. From the test, it was found that the magnitude of the error only varied between 1.34° and 6.22°, which can be considered to be small, as the average error is less than 3.5%. Thus, the close relationship between the SC cues and the elevation angle will allow for the vertical direction of the source to be robustly localized.

### 3.5. Binaural Localization Strategy

In summary, the direction of the sound source on the azimuth plane can be calculated using the ITD cue via cross correlation on the incident signals. The resulting AoA can then be used in order to estimate the value of θ. To predict the actual distance of the source from the DUT, the properties from the SPL cues can be exploited. Nevertheless, due to the structure of the head and the 3D-printed ears, estimations via SPL are not sufficient, thus the estimation via $DRT_{60}$ auditory cue that has less variation against angles is needed together with the weighting parameter derived in the preceding section to remove ambiguous data points. With regard to the elevation angle, SC will be exploited by finding the amplitude and peak frequencies from the signal’s spectral components.

To improve the performance during real-time experiments, induced secondary cues are introduced based on the estimated distance and elevation angle, which are represented by η and μ, respectively. Specifically, η=1 when the sound source is estimated at the front side of the DUT (based on the SPL), and μ=1 when the estimated ϕ is within $\Omega_{f}$. Hence the parameter ϵ will be unity when both η and μ are one, which corresponds to Equation (12). This will be the first stage of the ambiguity elimination technique. To treat the front-rear confusion further on the resulting azimuth angle, the values of η and μ will be cross-checked at the second stage; i.e., if η=0 and μ=1, then the sound source is expected to be at the mirrored position along the interaural axis (i.e., front side). This was formulated based on the idea that prediction based on μ would be more accurate due to the small position errors that are presented in Section 3.4. However, exceptions are imposed for the border case where the estimated angle within the margin areas; i.e., (85°,95°) and (265°,275°) remain unchanged. The whole procedure for the binaural localization with ambiguity elimination partitioned into two stages is summarized in Algorithm 1. For clarity purposes, $\hat{\theta }$, $\hat{\phi }$, $\hat{d}$ will be used to denote the estimated values for θ, ϕ and *d*, respectively.
**Algorithm 1** Binaural Localization via Spatial Auditory Cues**Require:**
SPL, $DRT_{60}$, SC**Ensure:**$\hat{\theta },\hat{\phi },\hat{d}$ and x,y,z                ▹ Estimated coordinates1:**while** true **do**2:    **procedure** Distance Estimations(SPL, $DRT_{60}$)3:        $\{d_{R}\}\leftarrow$ Equation (5){α}4:        $\{d_{b},d_{f}\}\leftarrow$ Equation (6){α}5:    **end procedure**6:    **procedure** Azimuth Angle Encoding(SPL)7:        $\{\tau_{d}\}\leftarrow$ Equation (2)8:        {β}← Equation (1)9:        $\left\{\theta_{0}\right\}\leftarrow \left\{\beta \right\}$              ▹ Estimated θ (before correction)10:    **end procedure**11:    **procedure** Elevation Angle Encoding(SC)12:        $\{\phi_{i(i=1,\ldots ,6)}\}\leftarrow$ Equation (15)$\left\{A_{p},\alpha_{0}\right\}$13:        $\hat{\phi }=\left(1/6\right)\sum_{i=1}^{6}\left(\phi_{i}\right)$                   ▹ Estimated ϕ14:    **end procedure**15:    **procedure** Ambiguity Elimination($\hat{\phi },d_{b},d_{f},d_{R}$)16:        $d_{bR}=\left|d_{b}-d_{R}\right|$; $d_{fR}=\left|d_{f}-d_{R}\right|$17:        **if** $d_{fR}<d_{bR}$ **then**                     ▹ Stage 118:           η=1;19:        **else** η=0;20:        **end if**21:        **if** $\hat{\phi }\in \Omega_{f}$ **then**22:           μ←1;23:        **else**24:            μ←0;25:        **end if**26:        ϵ=μ×η;27:        $\{\hat{d}\}\leftarrow$ Equation (11)$\left\{\epsilon ,d_{f},d_{R}\right\}$            ▹ Estimated *d*28:        **if** (μ=1 and η=0) **then**                 ▹ Stage 229:           **if** ($0\leq \hat{\theta }\leq 85$) **then**30:               $\hat{\theta }\leftarrow 180{}^{\circ}-\theta_{0}$;             ▹ Mirrored angle (left side)31:           **else if** ($275\leq \hat{\theta }<360$) **then**32:               $\hat{\theta }\leftarrow 540{}^{\circ}-\theta_{0}$;           ▹ Mirrored angle (right side)33:           **end if**34:        **else**35:           $\hat{\theta }\leftarrow \theta_{0}$;36:        **end if**37:    **end procedure**38:    **procedure** Localization($\left(\hat{\theta },\hat{\phi },\hat{d}\right)$)  ▹ Polar to 3D Cartesian coordinates39:        $x=\hat{d}\sin\left(\hat{\phi }\right)\cos\left(\hat{\theta }\right)$; $y=\hat{d}\cos\left(\hat{\phi }\right)\cos\left(\hat{\theta }\right)$; $z=\hat{d}\cos\left(\hat{\phi }\right)$40:    **end procedure**41:**end while**

## 4. Experiments and Performance Evaluations

**Figure A1:**
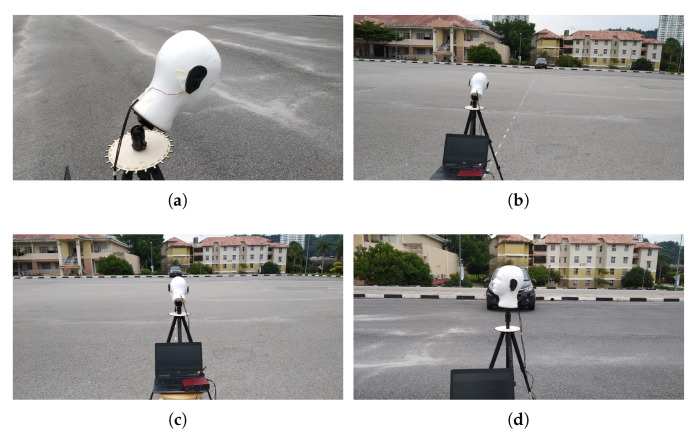
Experimental area and setup; (**a**) Illustration on how the DUT was rotated to estimate different values of ϕ; (**b**) Illustration on the test for (d,θ,ϕ)=(13,0°,45°); (**c**) Illustration on the test for (d,θ,ϕ)=(19,0°,45°); (**d**) Illustration on the test for (d,θ,ϕ)=(6,270°,0°).

This section presents the results from real-time experiments when the sound source was placed at 30 different locations in the 3D space. The tests were conducted in a car park area with the model being placed on the road, as shown in Figure A1 (in Appendix A), which has existing linear markers that allow for accurate distance and direction measurements. Three different distances i.e., d=6 m, d=13 m, and d=19 m, with various sets of θ and ϕ were randomly selected for performance evaluations. Without a loss of generality, measurements for θ and ϕ were taken by rotating the receiver instead of the sound source, as it was relatively easier to control.

**Figure 18:**
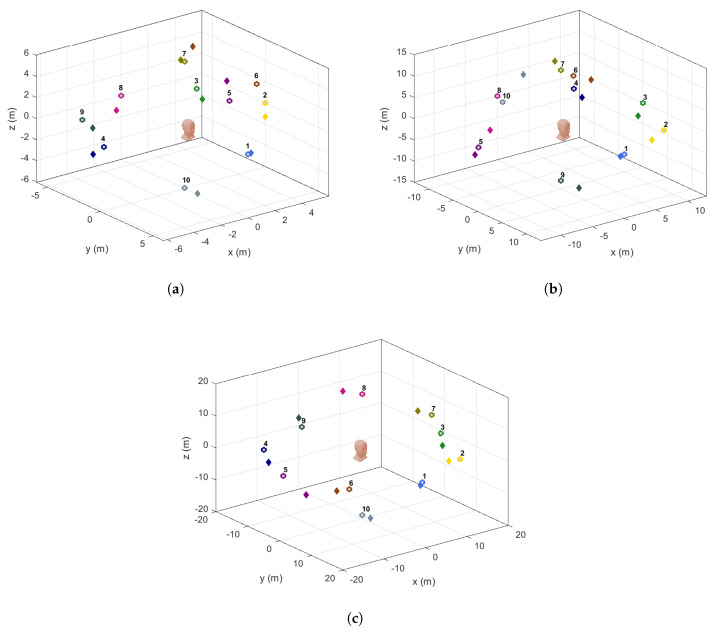
Illustrations on the actual positions (denoted by the colored circles) and the corresponding estimated positions (denoted by ’diamonds’ of the same color) in a 3D space when the sound source was placed at (**a**) 6 m; (**b**) 13 m; and (**c**) 19 m, away from the DUT (represented by the head icon). All of the positions considered were correctly localized with small position errors, except for one point found in (**a**) (at k=6), which was a result from the ambiguity issue.

The values for $\hat{d},\hat{\theta }$ and $\hat{\phi }$ when Algorithm 1 was applied are presented in Table 2, which have been partitioned according to the values of *d*. All of the captured data, including the secondary cues, η,μ and ϵ that were used for ambiguity elimination can be referred in Table A3 in the Appendix A. For clarity purposes, the variable *k* is used in order to represent the experiment number for each distance considered. Figure 18 shows the estimated and actual locations of the sound source with respect to the DUT in a 3D Cartesian plane that have also been plotted according to the values of *d*, i.e., (a) d=6 m, (b) d=13 m, and (c) d=19 m. The actual coordinates are represented by the colored circles, while the corresponding predicted coordinates are represented by the “diamonds” of the same color. The numbers next to the circles are included to denote the values of *k* from Table 2. By observing the plots, all of the coordinates considered were correctly localized with small position errors, except for k=6 in (a). This was caused by the value of η which was supposed to be 1 instead of 0, hence the estimated azimuth was interpreted at the mirrored position of the captured angle, which explains the large difference. Nevertheless, when comparing with the results without the application of Algorithm 1 from Table 3 (complete individual data in Table A3), we can see that the total number of ambiguous points (AP) is 9. This demonstrates that the proposed method has significantly reduced the total number of AP.

**Figure 19:**
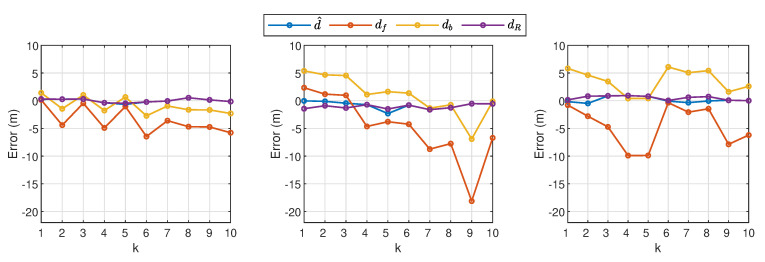
Distance error based on $d_{f}$, $d_{b}$, $d_{R}$, and $\hat{d}$ when d=6 m (left plot), d=13 m (middle plot), and d=19 m (right plot) from the 30 experiments. The distance estimation via application of Algorithm 1 is represented by the blue line.

In order to evaluate the localization performance, the following errors are defined:

(16) $$\begin{array}{c} e\left(j\right)=j-\hat{j};\;j=d,\theta ,\phi \end{array}$$

which calculates the deviation of the estimated from the actual values, and

(17) $$\begin{array}{c} E_{av}\left(j\right)=\frac{1}{10}\sum_{k=1}^{10}\left|e_{k}\left(j\right)\right|;\;j=d,\theta ,\phi \end{array}$$

which is the average value of absolute errors. Figure 19 shows the plots of e(d) (represented by the blue line), which is also compared against the corresponding errors when $d_{b}$, $d_{f}$, and $d_{R}$ are used as the estimated distance. From the plot, it is observed that the proposed method has successfully kept the error minimum for all experiments when compared to the performance by the other three methods.

**Figure 20:**
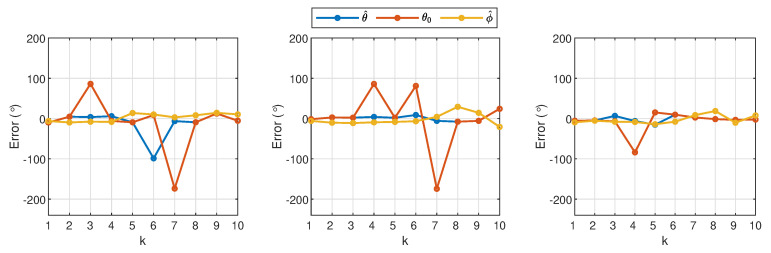
Errors in the azimuth and elevation planes when d=6 m (**left plot**), d=13 m (**middle plot**), and d=19 m (**right plot**) from the 30 experiments. The estimated azimuth angles with and without application of Algorithm 1 are represented by the blue and orange lines, respectively. The yellow line corresponds to the estimated elevation angle, which is consistently close to zero from all experiments.

With regard to the accuracy of the estimated angles, Figure 20, which shows the plots for e(θ) and e(ϕ), is also compared against the error before the azimuth angle was amended in Stage 2 of Algorithm 1, i.e., $\theta_{0}$. The large peaks shown from the orange plots correspond to the results from the ambiguous data points where the mirrored positions of the source were not corrected using the secondary cues from the proposed method. Other than that, it is observed that ε(ϕ) is consistently close to zero for all experiments, which has also become the contributing factor for the success in the ambiguity elimination technique. The overall average errors from both figures are summarized in Table 3 where ${\tilde{E}}_{av}=\left(E_{av,d=6}+E_{av,d=13}+E_{av,d=19}\right)/3$. From the data presented, the proposed method has significantly improved the performance by reducing the errors in distance and angle estimations. It is also worth noting that, without the $DRT_{60}$ and SC measurements as well as the secondary cues, the estimated sound source locations on the azimuth plane would be 100% ambiguous. In particular, with only Stage 1 in Algorithm 1, which also heavily relies on the ITD method (refer to $\theta_{0}$ in Table 3), the total ambiguous points (AP) was reduced to 30%, but, when combined with Stage 2 (refer to $\hat{\theta }$ in Table 3), the total AP has been considerably reduced to 3.3%. Table 3 also shows that, due to the large number of AP from $\theta_{0}$, the average error, ${\tilde{E}}_{av}$, is approximately 28.3°, which is significantly higher than that when the complete Algorithm 1 is applied, which only gives an average error of 9.6°.

## 5. Discussion

The results, as presented in Table 3, have demonstrated significant improvements in the distance and angle estimations, thus showing that using PLA-based 3D printed ears is practical, particularly for front-rear disambiguation in outdoor environments. While this might work in several other environments, modifications on the strategy may be needed if there is a sudden or drastic change in the acoustic scene. Thus, to detect as well as identify the changes, machine learning can be used and the resulting mechanism can be embedded into the system so as to ensure the proposed strategy is adaptive to the changes. Apart from that, as the reverberation properties in outdoor spaces can be modeled according to the sound source frequency as well as the nature of the spaces, the $DRT_{60}$-based distance estimation technique in Section 3.2 can always be tuned in order to make it applicable to other environments.

## 6. Conclusions and Future Work

This paper contributes its findings to binaural localization using auditory cues. Instead of using a HATS (this costs approximately USD20k, and USD120 for daily rent) or an ear simulator, this work uses a pair of cheap PLA-based 3D-printed ears with mechanical acoustic dampers and filters covering the microphones. The analysis that was obtained from this work shows that there is a possibility in using cheap 3D-printed materials in order to simulate an actual ear. Other benefits of using a 3D printed ear include the ability to quickly replicate this work, and to make modifications to the existing design to study how different shapes would affect the result.

From the conducted experiments, it has been demonstrated that the proposed strategy can considerably improve the binaural localization performance with average errors of 0.5 m for distance, 9.6° for azimuth angle, 10.3° for elevation angle, and, most importantly, a significant reduction of total ambiguous points to 3.3%. The results also reveal that the proposed model and methodology can provide a promising framework for further enhancement of binaural localization strategy.

Having dynamic cues, in addition to what this work has presented, can help enhance the accuracy, particularly when there is a drastic change in the acoustic scene or when the targeted sound source is moving. Tracking a moving source or multiple sources is significantly more complex, as Doppler effects come into play and, thus, the spectral cues has to account for the phenomena. Dynamic cues are useful to help further improve how the receiver perceives sound by essentially getting more sets of data. As discussed in Section 5, the method can be paired with advance algorithms in future works, such as deep learning, to help improve the detection of acoustic cues that are based on different situations.

## Figures and Tables

**Table 1:** DreamMaker OverLord printing parameters.

Parameter	Descriptions
Slicer	Cura 15.04.6
Material	PLA
Layer height	0.15 mm
Shell thickness	0.8 mm
Enable extraction	Yes
Bottom Thickness	0.6 mm
Fill density	100%
Print speed	60 mm/s
Nozzle Temperature	210 C
Nozzle size	0.4 mm
Layer thickness	0.1 mm
Extrusion overlap	0.15 mm
Travel speed	100 mm/s
Bottom layer speed	20 mm/s
Outer shell speed	50 mm/s
Inner shell speed	60 mm/s
Minimal layer time	5 s

**Table 2:** Numerical Results.

	d=6 m					d=13 m					d=19 m				
	**Actual**		**Estimated**			**Actual**		**Estimated**			**Actual**		**Estimated**		
k	θ(°)	ϕ(°)	$\hat{d}\left(m\right)$	$\hat{\theta }\left({}^{\circ}\right)$	$\hat{\phi }\left({}^{\circ}\right)$	θ(°)	ϕ(°)	$\hat{d}\left(m\right)$	$\hat{\theta }\left({}^{\circ}\right)$	$\hat{\phi }\left({}^{\circ}\right)$	θ(°)	ϕ(°)	$\hat{d}\left(m\right)$	$\hat{\theta }\left({}^{\circ}\right)$	$\hat{\phi }\left({}^{\circ}\right)$
1	0	0	5.75	9.65	6.21	0	0	13.03	1.54	5.81	0	0	19.17	5.42	9.25
2	90	0	5.73	85.35	9.55	45	0	13.12	42.15	10.22	45	0	19.49	49.62	5.32
3	135	0	5.69	131.22	7.65	90	0	13.45	87.66	11.12	90	0	18.13	83.32	7.93
4	270	0	6.38	264.12	8.22	135	0	13.72	130.9	9.25	225	0	18.08	231.12	8.39
5	0	45	6.67	9.21	31.21	270	0	15.32	267.96	8.22	270	0	18.22	285.22	14.07
6	45	45	6.23	143.68	34.79	135	15	13.8	126.12	21.57	315	0	19.08	305.32	7.7
7	0	90	6.06	6.31	86.9	0	90	14.62	5.69	85.7	45	45	19.35	42.32	36.32
8	0	180	5.47	9.16	171.88	0	180	14.27	7.66	150.66	0	90	19.05	1.35	71.31
9	225	180	5.86	212.46	165.68	0	270	13.54	5.69	255.66	0	180	18.92	3.22	190.21
10	0	270	6.15	5.35	259.63	270	135	13.57	245.69	155.69	0	270	18.99	2.32	262.32

**Table 3:** Performance evaluations in terms of errors and total number of ambiguous points (AP).

	Distance Error					Angle Error		
**Index**	$d_{f}$ **(SPL)**	$d_{b}$ **(SPL)**	$d_{R}$ (${\mathrm{DRT}}_{60}$)	$\hat{d}$ **(Alg. 1)**	**Index**	$\hat{\theta }$ **(Alg. 1)**	$\theta_{0}$	$\hat{\phi }$ **(SC)**
					${\tilde{E}}_{av}$(°)	9.59	28.3	10.3
${\tilde{E}}_{av}$ (m)	4.7	2.6	0.6	0.5	Total AP	1	9	0
					Total AP (%)	3.3	30	0

## Supplementary Materials

The following are available online at https://www.mdpi.com/1424-8220/21/1/227/s1.

## Notations and Acronyms

The following notations and acronyms are used in this manuscript:

**Notations/ Acronyms**	**Descriptions**
PLA	Polylactic Acid
HATS	Head and Torso Simulator
HRTF	Head Related Transfer Function
DRR	direct-to-reverberant ratio
SPL, ILD, ITD	sound pressure level, interaural level difference, interaural time difference
AoA	Angle of Arrival
$f_{s}$	Sampling rate
Mic L, Mic R	Left microphone, right microphone
RT	reverberation time
$DRT_{60}$	the estimated time for the combined direct and reverberant energy decay curve to drop by 60 dB
SC	spectral cues
ADC	analog to digital converter
DUT	device under test
FFT	Fast Fourier Transform
dB, dBFS	decibels/decibels relative to full scale
θ,ϕ,*d*	actual azimuth angle, elevation angle, Euclidean distance of the sound source from the DUT
$\hat{\theta }$,$\hat{\phi }$,$\hat{d}$	estimated azimuth angle, elevation angle, Euclidean distance of the sound source from the DUT
β	notation for AoA
$\theta_{0}$	Estimated azimuth angle before correction
μ,η,ϵ	induced secondary cues (as described in Algorithm 1)
x,y,z	estimated coordinates of the sound source in the 3D space
$\tau_{d}$	notation for ITD
$d_{f}$, $d_{b}$	estimated distance based on SPL regression curve when the sound source is in the front/back of the receiver
$d_{R}$	estimated distance based on $DRT_{60}$
$\tau_{R}$	notation for $DRT_{60}$ (in milliseconds)
$\Omega_{f}$, $\Omega_{b}$	sets of elevation angle defined in Equation (7)
ν	weighting parameter for the estimated distance
*e*	deviation of the estimated from the actual values (for θ,ϕ, and *d*)
$E_{cum}$, $E_{av}$	Cumulative error, Average of absolute error
R	fields of real numbers
$R^{+}$,$R^{-}$	fields of positive real numbers, fields of negative real numbers

## Appendix A

**Table A1:** Datasets for the analysis in Section 3.2.

	$\phi \in \Omega_{f}$								$\phi \in \Omega_{b}$							
k	1	2	3	4	5	6	7	8	1	2	3	4	5	6	7	8
*d*	6	6	8	10	10	12	16	19	6	6	8	10	10	12	16	19
ϕ	0	0	15	30	30	45	80	80	110	110	135	180	180	180	260	260
θ	0	45	90	270	135	0	0	270	0	45	90	270	135	0	0	270

**Table A2:** Absolute errors from the estimated distance for all experiments. Note that SPL and $DRT_{60}$ methods represent the individual components of the proposed method (Algorithm 1).

	d = 6										d = 13										d = 19									
k	**1**	**2**	**3**	**4**	**5**	**6**	**7**	**8**	**9**	**10**	**1**	**2**	**3**	**4**	**5**	**6**	**7**	**8**	**9**	**10**	**1**	**2**	**3**	**4**	**5**	**6**	**7**	**8**	**9**	**10**
SPL ($d_{f}$)	0.14	4.41	0.46	4.92	1.04	6.47	3.6	4.69	4.74	5.78	2.34	1.18	0.97	4.65	3.8	4.24	8.74	7.75	18.1	6.71	0.79	2.8	4.73	9.91	9.89	0.36	2.06	1.46	7.87	6.2
SPL ($d_{b}$)	1.42	1.46	1.03	1.77	0.66	2.73	0.96	1.64	1.67	2.31	5.39	4.67	4.54	1.12	1.63	1.36	1.34	0.75	6.92	0.12	5.82	4.62	3.47	0.4	0.41	6.09	5.06	5.42	1.6	2.59
$DRT_{60}$	0.3	0.27	0.31	0.38	0.47	0.23	0.06	0.53	0.14	0.15	1.45	0.91	1.31	0.72	1.5	0.8	1.62	1.27	0.54	0.57	0.14	0.81	0.87	0.92	0.78	0.04	0.6	0.73	0.08	0.01
Algorithm 1	0.25	0.27	0.31	0.38	0.67	0.23	0.06	0.53	0.14	0.15	0.03	0.12	0.45	0.72	2.32	0.8	1.62	1.27	0.54	0.57	0.17	0.49	0.87	0.92	0.78	0.08	0.35	0.05	0.08	0.01

**Table A3:** The estimated azimuth angles, elevation angles, secondary cues and ambiguous points (AP) for each experiment. The first, second and third tables from the top correspond to the data when d=6 m, d=13 m, and d=19 m respectively.

	k	1	2	3	4	5	6	7	8	9	10
	Actual azimuth θ	0	90	225	270	0	45	0	0	225	0
Estimated azimuth	$\theta_{0}$	9.65	85.4	48.8	276	9.21	36.3	173.4	9.16	212.5	5.35
	$\hat{\theta }$ (Alg. 1)	9.65	85.4	131.2	264	9.21	143.7	6.31	9.16	212.5	5.35
Estimated elevation	$\hat{\phi }$	6.2	9.6	7.7	8.22	31.2	34.8	86.9	172	166	260
Secondary cues	μ	1	1	1	1	1	1	1	0	0	0
	η	1	0	0	0	1	0	0	0	0	0
	ϵ	1	0	0	0	1	0	0	0	0	0
AP	Without Alg. 1	0	0	1	1	0	0	1	0	0	0
	With Alg. 1	0	0	0	0	0	1	0	0	0	0
	Actual azimuth θ	0	45	90	135	270	135	0	0	0	270
Estimated azimuth	$\theta_{0}$	1.54	42.2	87.7	49.1	268	53.9	174.3	7.66	5.69	246
	$\hat{\theta }$ (Alg. 1)	1.54	42.2	87.7	131	268	126	5.69	7.66	5.69	246
Estimated elevation	$\hat{\phi }$	5.81	10.2	11.1	9.25	8.22	21.6	85.7	151	256	156
Secondary cues	μ	1	1	1	1	1	1	1	0	0	0
	η	1	1	1	0	1	0	0	0	0	0
	ϵ	1	1	1	0	1	0	0	0	0	0
AP	Without Alg. 1	0	0	0	1	0	1	1	0	0	0
	With Alg. 1	0	0	0	0	0	0	0	0	0	0
	Actual azimuth θ	0	45	90	225	270	315	45	0	0	0
Estimated azimuth	$\theta_{0}$	5.42	49.6	96.7	309	255	305	42.32	1.35	3.22	2.32
	$\hat{\theta }$ (Alg. 1)	5.42	49.6	83.3	231	285	305	42.32	1.35	3.22	2.32
Estimated elevation	$\hat{\phi }$	9.25	5.32	7.93	8.39	14.1	7.7	36.32	71.3	190	262
Secondary cues	μ	1	1	1	1	1	1	1	1	0	0
	η	1	1	0	0	0	1	1	1	0	0
	ϵ	1	1	0	0	0	1	1	1	0	0
AP	Without Alg. 1	0	0	1	1	1	0	0	0	0	0
	With Alg. 1	0	0	0	0	0	0	0	0	0	0

## References

[1] Argentieri S., Danès P., Souères P. (2015). A survey on sound source localization in robotics: From binaural to array processing methods. Comput. Speech Lang..

[2] Zhong X., Sun L., Yost W. (2016). Active Binaural Localization of Multiple Sound Sources. Robot. Auton. Syst..

[3] Kumpik D.P., Campbell C., Schnupp J.W.H., King A.J. (2019). Re-weighting of Sound Localization Cues by Audiovisual Training. Front. Neurosci..

[4] Zhang P., Hartmann W. (2009). On the ability of human listeners to distinguish between front and back. Hear. Res..

[5] Paul S. (2009). Binaural Recording Technology: A Historical Review and Possible Future Developments. Acta Acust. United Acust..

[6] Zhang W., Samarasinghe P.N., Chen H., Abhayapala T.D. (2017). Surround by Sound: A Review of Spatial Audio Recording and Reproduction. Appl. Sci..

[7] Yang Y., Chu Z., Shen L., Xu Z. (2016). Functional delay and sum beamforming for three-dimensional acoustic source identification with solid spherical arrays. J. Sound Vib..

[8] Fischer B.J., Seidl A.H. (2014). Resolution of interaural time differences in the avian sound localization circuit—A modeling study. Front. Comput. Neurosci..

[9] Du R., Liu J., Zhou D., Meng G. (2018). Adaptive Kalman filter enhanced with spectrum analysis to estimate guidance law parameters with unknown prior statistics. Proc. Inst. Mech. Eng. Part G J. Aerosp. Eng..

[10] Dorman M., Loiselle L., Stohl J., Yost W., Spahr T., Brown C., Natale S. (2014). Interaural Level Differences and Sound Source Localization for Bilateral Cochlear Implant Patients. Ear Hear..

[11] Fischer R., Weber J. Real World Assessment of Auditory Localization Using Hearing Aids. https://www.audiologyonline.com/articles/real-world-assessment-of-auditory-localization-~using-hearing-aids-11719.

[12] Spagnol S. (2015). On distance dependence of pinna spectral patterns in head-related transfer functions. J. Acoust. Soc. Am..

[13] Ahveninen J., Kopco N., Jääskeläinen I. (2013). Psychophysics and Neuronal Bases of Sound Localization in Humans. Hear. Res..

[14] Risoud M., Jean Noel H., Gauvrit F., Renard C., Bonne N.X., Vincent C. (2019). Azimuthal sound source localization of various sound stimuli under different conditions. Eur. Ann. Otorhinolaryngol. Head Neck Dis..

[15] Zhong X.L., Xie B.S. (2014). Head-Related Transfer Functions and Virtual Auditory Display. Soundscape Semiot. Localization Categ..

[16] Kim E., Nakadai K., Okuno H. (2014). Improved sound source localization in horizontal plane for binaural robot audition. Appl. Intell..

[17] Georganti E., Mourjopoulos J. Statistical relationships of Room Transfer Functions and Signals. Proceedings of the Forum Acusticum.

[18] Lovedee-Turner M., Murphy D. (2018). Application of Machine Learning for the Spatial Analysis of Binaural Room Impulse Responses. Appl. Sci..

[19] Ding J., Ke Y., Cheng L., Zheng C., Li X. (2020). Joint estimation of binaural distance and azimuth by exploiting deep neural networks. J. Acoust. Soc. Am..

[20] Pang C., Liu H., Zhang J., Li X. (2017). Binaural Sound Localization Based on Reverberation Weighting and Generalized Parametric Mapping. IEEE/ACM Trans. Audio Speech Lang. Process..

[21] Larsen E., Iyer N., Lansing C., Feng A. (2008). On the minimum audible difference in direct-to-reverberant energy ratio. J. Acoust. Soc. Am..

[22] Garas J., Sommen P. Improving virtual sound source robustness using multiresolution spectral analysis and synthesis. Proceedings of the Audio Engineering Society Convention 105.

[23] Iida K. (2019). Head-Related Transfer Function and Acoustic Virtual Reality.

[24] Fingerhuth S., Bravo J.L., Bustamante M., Pizarro F. (2020). Experimental Study of the Transfer Function of Replicas of Pinnae of Individuals Manufactured with Alginate. IEEE Lat. Am. Trans..

[25] Rodemann T., Ince G., Joublin F., Goerick C. Using binaural and spectral cues for azimuth and elevation localization. Proceedings of the 2008 IEEE/RSJ International Conference on Intelligent Robots and Systems.

[26] Heffner R., Koay G., Heffner H. (2010). Use of binaural cues for sound localization in large and small non-echolocating bats: Eidolon helvum and Cynopterus brachyotis. J. Acoust. Soc. Am..

[27] Schillebeeckx F., Mey F.D., Vanderelst D., Peremans H. (2011). Biomimetic Sonar: Binaural 3D Localization using Artificial Bat Pinnae. Int. J. Robot. Res..

[28] Odo W., Kimoto D., Kumon M., Furukawa T. (2017). Active Sound Source Localization by Pinnae with Recursive Bayesian Estimation. J. Robot. Mechatron..

[29] Grothe B., Pecka M. (2014). The natural history of sound localization in mammals—A story of neuronal inhibition. Front. Neural Circuits.

[30] Heffner H., Heffner R. (2018). The evolution of mammalian hearing. AIP Conf. Proc..

[31] Kulaib A., Al-Mualla M., Vernon D. (2009). 2D Binaural Sound Localization: For Urban Search and Rescue Robotics. Mob. Robot. Solut. Chall..

[32] Rascon C., Meza I. (2017). Localization of sound sources in robotics: A review. Robot. Auton. Syst..

[33] Kerzel M., Strahl E., Magg S., Navarro-Guerrero N., Heinrich S., Wermter S. NICO—Neuro-Inspired COmpanion: A Developmental Humanoid Robot Platform for Multimodal Interaction. Proceedings of the 2017 26th IEEE International Symposium on Robot and Human Interactive Communication (RO-MAN).

[34] Deshpande N., Braasch J. (2019). Detection of early reflections from a binaural activity map using neural networks. J. Acoust. Soc. Am..

[35] Wang M., Zhang X.L., Rahardja S. (2020). An Unsupervised Deep Learning System for Acoustic Scene Analysis. Appl. Sci..

[36] Argentieri S., Portello A., Bernard M., Danès P., Gas B., Blauert J. (2013). Binaural Systems in Robotics. The Technology of Binaural Listening.

[37] Ma N., Gonzalez J., Brown G. (2018). Robust Binaural Localization of a Target Sound Source by Combining Spectral Source Models and Deep Neural Networks. IEEE/ACM Trans. Audio Speech Lang. Process..

[38] Scharine A., Letowski T., Sampson J. (2009). Auditory situation awareness in urban operations. J. Mil. Strateg. Stud..

[39] Sebastian Mannoor M., Jiang Z., James T., Kong Y., Malatesta K., Soboyejo W., Verma N., Gracias D., McAlpine M. (2013). 3D Printed Bionic Ears. Nano Lett..

[40] Gala D., Lindsay N., Sun L. (2019). Realtime Active Sound Source Localization for Unmanned Ground Robots Using a Self-Rotational Bi-Microphone Array. J. Intell. Robot. Syst..

[41] Magassouba A., Bertin N., Chaumette F. (2018). Aural Servo: Sensor-Based Control From Robot Audition. IEEE Trans. Robot..

[42] Zohourian M., Martin R. (2020). Binaural Direct-to-Reverberant Energy Ratio and Speaker Distance Estimation. IEEE/ACM Trans. Audio Speech Lang. Process..

[43] Lu Y.C., Cooke M. Binaural distance perception based on direct-to-reverberant energy ratio. Proceedings of the International Workshop on Acoustic Echo and Noise Control.

[44] Thomas P., Van Renterghem T., De Boeck E., Dragonetti L., Botteldooren D. (2013). Reverberation-based urban street sound level prediction. J. Acoust. Soc. Am..

[45] Yang H.S., Kang J., Kim M.J. (2017). An experimental study on the acoustic characteristics of outdoor spaces surrounded by multi-residential buildings. Appl. Acoust..

